# Temporal trends in inpatient care use for adult mental disorders in Czechia: a nationwide register-based study from 1994 to 2015

**DOI:** 10.1007/s00127-024-02691-5

**Published:** 2024-05-31

**Authors:** Libor Potočár, Petr Winkler, Pavel Mohr, Tomáš Formánek

**Affiliations:** 1 Department of Public Mental Health, National Institute of Mental Health, Klecany, Czechia; 2 PROMENTA Research Center, Department of Psychology, University of Oslo, Oslo, Norway; 3 Health Service and Population Research Department, Institute of Psychiatry, Psychology and Neuroscience, King’s College London, London, UK; 4 Clinical Center, National Institute of Mental Health, Klecany, Czechia; 5 Third Faculty of Medicine, Charles University, Prague, Czechia; 6 Department of Psychiatry, University of Cambridge, Cambridge, UK

**Keywords:** Psychiatric hospitalization, Hospital-based care, Healthcare services, Common mental disorders, Schizophrenia, Substance use disorders, Temporal trends, National data, Central and Eastern Europe

## Abstract

**Purpose:**

To describe temporal trends in inpatient care use for adult mental disorders in Czechia from 1994 until 2015.

**Methods:**

Data from the nationwide register of inpatient care use and yearly census data were used to calculate (a) yearly admissions rates, (b) median length of stay, and (c) standardized inpatient-years for adult mental disorders (ICD-10 codes F0-F6] or G30). Segmented regressions were used to analyze age- and sex-specific temporal trends.

**Results:**

Admission rates were increasing in adults (average annual percent change = 0.51; 95% confidence interval = 0.16 to 0.86 for females and 1.01; 0.63 to 1.40 for males) and adolescents and emerging adults (3.27; 2.57 to 3.97 for females and 2.98; 2.08 to 3.88 for males), whereas in seniors, the trend was stable (1.22; -0.31 to 2.73 for females and 1.35; -0.30 to 2.98 for males). The median length of stay for studied mental disorders decreased across all age and sex strata except for a stable trend in male adolescents and emerging adults (-0.96; -2.02 to 0.10). Standardized inpatient-years were decreasing in adults of both sexes (-0.85; -1.42 to -0.28 for females and -0.87; -1.19 to -0.56 for males), increasing in female adolescents and emerging adults (0.95; 0.42 to 1.47), and stable in the remaining strata.

**Conclusion:**

Psychiatric hospital admissions were increasing or stable coupled with considerable reductions in median length of stay, suggesting that inpatient episodes for adult mental disorders have become more frequent and shorter over time. The overall psychiatric inpatient care use was decreasing or stable in adults and seniors, potentially implying a gradual shift away from hospital-based care.

**Supplementary Information:**

The online version contains supplementary material available at 10.1007/s00127-024-02691-5.

## Introduction

Mental disorders are associated with premature mortality [1, 2] and account for up to 19% of all years lived with disability [3, 4]. Moreover, while disability-adjusted life years of all diseases have been continuously decreasing between 1990 and 2016, these rates increased for mental and substance use disorders (SUD) by 4.3% and 12.0%, respectively [4]. As mental health-related disability is becoming an increasingly large component of disease burden [3, 5] and mental disorders lead to disproportionally high indirect economic costs [6], policymakers around the globe are facing challenges in mental health care provision.

## Psychiatric reforms and deinstitutionalization efforts

Sparked by the human rights movement [7–9] and facilitated by advances in psychopharmacological treatment [8], psychiatric care has been experiencing a major reform since the second half of the twentieth century. In particular, there has been a growing trend of implementing the policy of deinstitutionalization, that is, shifting the locus of mental healthcare away from large psychiatric hospitals towards community-based alternatives [10–16]. These new arrangements encompass a broad set of services such as day clinics, mobile crisis intervention teams, supported housing, and sheltered employment. Importantly, they can be seen as more cost-effective [13, 17–21], partially due to their role in intervening or delaying relapse or reducing the risk of re-admission to more costly inpatient care [17, 18, 22–24]. Deinstitutionalized mental healthcare systems also seem to deliver benefits related to wider recovery outcomes, including improved social functioning, quality of life, autonomy [25–27], employment [20, 28, 29], and settled housing and physical health [18], all while evidently not contributing to adverse outcomes such as criminality, homelessness or suicides [30].

In most Western countries, there has been a gradual shift in inpatient care provision characterized by stable or increasing hospital admission rates coupled with substantial decreases in the length of stay and lowering the number of psychiatric beds [31–37]. In other words, over time, fewer patients receive long-term treatment, but the number of short-term inpatient care episodes tends to stay stable or increase in many countries. However, the majority of public mental health research in Europe is carried out in Western countries [38], and the results from previous research are bound to a specific cultural context, socioeconomic conditions, and mental healthcare systems. Evidence on time trends in inpatient services usage in post-communist Central and Eastern Europe (CEE), including Czechia, is lacking.

## The Czech healthcare system

Mental health care in CEE is characterized by the persistence of large psychiatric hospitals, whereas most psychiatric reforms remain on the aspirational level [39–41]. In Czechia, 56.3% and 61.1% of the mental health budget were allocated to inpatient care in 2006 and 2015, respectively, while only 15.3% and 14% were to outpatient care [42–44]. In 2015, 64.5% of overall admissions related to mental disorders were handled in psychiatric hospitals and 32.2% in psychiatric wards of general hospitals [45].

However, Czechia launched its national mental health reform in 2013, seeking, among other priorities, to transform psychiatric hospitals and to decrease the number of long-term hospitalizations [40, 46]. Monitoring of health care services use is essential for evidence-based planning and implementation of health policies, and the future evaluation of the mental health reform warrants robust evidence on the pre-reform time period.

## The present study

In the present study, our objective was to describe time trends in inpatient care use for mental disorders in Czechia from 1994 until 2015. We aimed to describe the age- and sex-specific time development in (a) yearly admission rates, (b) length of stay, and (c) standardized inpatient-years in most commonly occurring adult mental disorders. Since this study was conceived as descriptive, we refrained from formulating any hypotheses, and we did not perform any hypothesis testing.

## Methods

### Data

We used data from the nationwide register of inpatient care use maintained by the Czech Institute of Health Information and Statistics (IHIS). This register contains routinely collected data on all-cause inpatient discharges in the Czech healthcare system starting from 1994. There are approximately 2.3 million hospital records for each calendar year, out of which approximately 2.5% (nearly 60,000) are related to mental disorders. The provision of healthcare in Czechia is based on the compulsory insurance model, with the population coverage being virtually universal [47].

Data are recorded on a standard form that is obligatorily filled out by health professionals upon a patient’s discharge, and then sent directly to the IHIS. This database contains basic socio-demographic and key clinical information about patients, including date of admission and discharge and diagnoses based on the International Statistical Classification of Diseases 10^th^ Revision (ICD-10). Although there are no formal reports on the reliability and validity of diagnoses in the register, nationwide use of the ICD-10 taxonomy and health professionals’ training in the taxonomy should guarantee reasonably high-quality data. Moreover, diagnostic standards encompass psychopathological and physical assessments and psychological testing in multi-professional teams upon patient’s admission; therefore, diagnostic assessment is generally thorough.

To calculate admission rates and standardized inpatient-years, we obtained yearly census data broken down by five-year age groups and sex, describing Czech and foreign citizens permanently residing in Czechia on 1^st ^July of the respective calendar year, from the Czech Statistical Office.

### Study population

The study population included all individuals aged 15 or more years who received inpatient treatment for mental disorders in Czechia in the time period from 1^st^ January 1994 to 31^st^ December 2015. We used ICD-10 codes to identify records listing (a) any mental disorder (F0-F6 or G30), (b) dementia in Alzheimer’s disease (F00 or G30), (c) alcohol use disorders (AUD; F10), (d) drug use disorders (DUD; F11-F19), (e) schizophrenia (F20), (f) depression (F32-F33), (g) anxiety disorders (F4), (h) eating disorders (F50), and (i) specific personality disorders (F60) as the primary diagnosis. We selected these since they are among the most commonly occurring adult mental disorders and to facilitate comparability with previous studies of similar focus and scope [48]. When an individual was hospitalized multiple times in the same year for a given diagnosis, we counted each occurrence separately. In order to achieve a good balance between internal consistency and tractability of analyses, we decided to stratify the population per three age groups (adolescents and emerging adults (15–29 years [49]), adults (30–64 years), and seniors (65 + years)) and sex (males and females), leading to a total of 6 age- and sex-specific subpopulations.

### Outcomes

We investigated three outcomes: (a) admission rates, (b) median length of stay (LOS), and (c) standardized inpatient-years (SIY; also known as treatment prevalence [32]). Admission rates refer to the number of admitted individuals divided by the number of individuals at risk in each calendar year. To obtain the admission rates, we summed the number of admissions occurring in a given year, starting from 1^st^ January 1994. Since yearly census data was available, the calculated admissions per 100,000 persons at risk can be interpreted as 100,000 person-years (PY).

Second, LOS refers to a period of continuous stay in inpatient care and it is calculated by subtracting the admission date from the discharge date. Stays exceeding one calendar year were assigned to the year in which they began (i.e., admission year). Typically, the distribution of individual-level LOS is positively skewed, with a small proportion of long-stay hospitalizations. Thus, to reflect temporal changes in typical values of LOS, we calculated the median LOS. In line with the definition of the Organisation for Economic Co-operation and Development (OECD) [50], we did not consider day cases with a length of stay equal to zero. To avoid extremely uncertain estimates biased by outliers, LOS were not analyzed for strata with rare occurrences (i.e., dementia in Alzheimer’s disease in adults and eating disorders and specific personality disorders in seniors).

Finally, we computed inpatient-years by summing up all treatment days per year. That is, if a stay exceeded the admission year, the treatment days of hospitalized individuals were assigned to multiple calendar years in which the stay occurred. From the health services use perspective, inpatient-years represent the most comprehensive indicator of the use of inpatient care, integrating the information on both the number of admissions and the length of hospital stay. We calculated standardized inpatient-years by dividing the yearly counts of inpatient-years by the number of the population at risk in each calendar year. Consistently with median LOS computation, we excluded day cases.

### Statistical analyses

We analyzed the temporal trends in all three outcomes using a segmented or piece-wise regression analysis approach. This analytical method enables to identify transition points or breakpoints and to partition the observed data into separate linear time segments [51], thus allowing to address the potential non-linearity in time trends. A breakpoint or join-point refers to a point at which these segments connect, representing a statistically significant change in the trend [51]. Importantly, assuming that the change in rates is constant over each time segment, but varies among different time partitions, this approach allows to informatively summarize the identified time trends by calculating the annual percent changes (APCs) for each time segment.

For each combination of sex, age group, and diagnosis, we fitted a separate model. Due to over-dispersion, we used negative binomial distribution. We fitted models with yearly counts of psychiatric admissions, median LOS, and yearly inpatient-years as the outcome, respectively. We used time as the exposure in the models. To account for potential shifts in the age composition of the population and changing population size during the observation period, we used log-transformed age-specific population estimates as the offset variable in the models for admissions and inpatient-years. Since we did not test any specific hypothesis, we implemented a data-driven approach that chooses the best-fitting model and the number of breakpoints via the Bayesian information criterion (BIC; [52]). To prevent over-fitting, we defined two as the maximum number of potential breakpoints. If no breakpoints were identified, the solution corresponds to a simple linear model. Based on these models, we computed APCs with 95% confidence intervals (95% CIs). To facilitate comparison between groups with different identified breakpoints and time segments, we also calculated average annual percent changes (AAPCs) as the sum of the slopes weighted by corresponding covariate sub-interval width [53].

We performed all statistical analyses and data pre-processing steps using the R software (version 4.2.2.) [54]. We used the R package *segmented* (version 1.6.2) [51] for segmented regression analysis.

## Results

Table 1 shows absolute and relative frequencies of admissions to psychiatric inpatient care by sex, age group, and diagnosis for the entire period of 1994 to 2015.

**Table 1:** Descriptive statistics of the studied population for the entire period of 1994 to 2015

Diagnosis	Sex, *n* (%)		Age category, *n* (%)		
	Males	Females	Adolescents & emerging adults	Adults	Seniors
Any mental disorder	818 272 (51.17)	780 980 (48.83)	349 798 (21.87)	884 082 (55.28)	337 261 (21.09)
Dementia in Alzheimer’s disease	19 756 (32.44)	41 143 (67.56)	NA	4 548 (7.47)	56 351 (92.53)
AUD	253 083 (70.11)	107 922 (29.89)	56 815 (15.74)	282 050 (78.13)	17 081 (4.73)
DUD	89 289 (65.99)	46 014 (34.01)	85 908 (63.49)	44 518 (32.90)	3 616 (2.67)
Schizophrenia	92 644 (59.51)	63 039 (40.49)	40 773 (26.19)	104 888 (67.37)	9 682 (6.22)
Depression	35 167 (32.30)	73 725 (67.70)	9 666 (8.88)	72 364 (66.45)	26 333 (24.18)
Anxiety disorders	105 362 (38.62)	167 442 (61.38)	70 776 (25.94)	157 628 (57.78)	30 715 (11.26)
Eating disorders	1 539 (8.73)	16 081 (91.27)	10 861 (61.64)	2 366 (13.43)	534 (3.03)
Specific personality disorders	19 083 (47.30)	21 264 (52.70)	18 173 (45.04)	20 310 (50.34)	1 443 (3.58)

The results are expressed as counts (n) with proportions (%). NA denotes situations when the given diagnosis was not considered in the particular stratum. “AUD” an “DUD” refer to alcohol use disorders and drug use disorders, respectively

### Admission rates

**Fig. 1:**
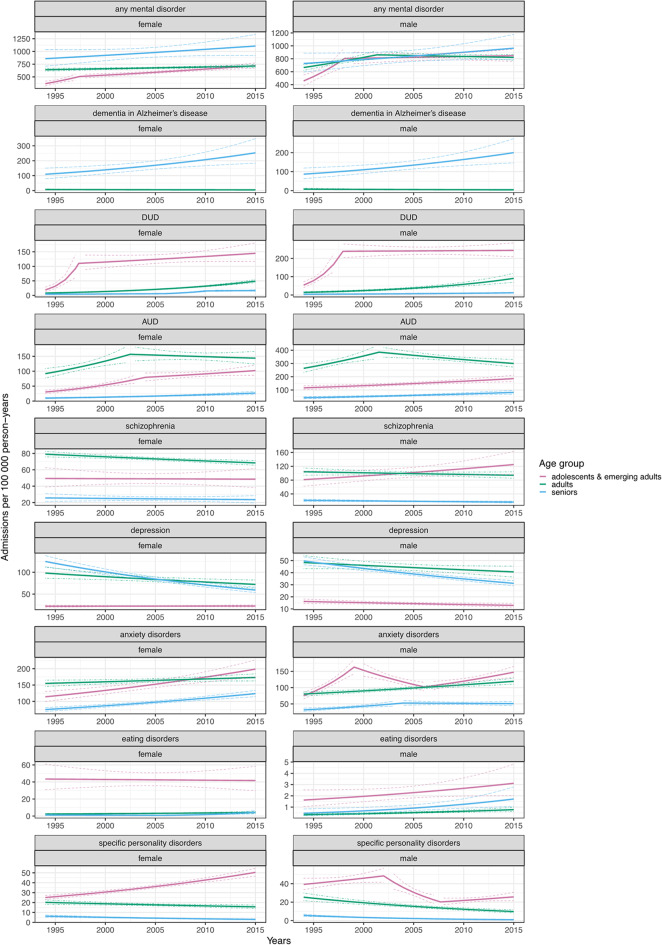
Age- and sex-specific temporal trends in admissions to inpatient care. The results are expressed as annual percent changes with 95% confidence intervals. “AUD” an “DUD” refer to alcohol use disorders and drug use disorders, respectively

When considering hospitalizations for any adult mental disorder, we detected increases in admissions in adults (AAPC = 0.51; 95% CI = 0.16 to 0.86 for females and 1.01; 0.63 to 1.40 for males) and adolescents and emerging adults (3.27; 2.57 to 3.97 for females and 2.98; 2.08 to 3.88 for males), whereas in seniors, the estimates were consistent with a stable trend (1.22; -0.31 to 2.73 for females and 1.35; -0.30 to 2.98 for males). For dementia in Alzheimer’s disease, we observed increases in seniors of both sexes (4.08; 1.45 to 6.55 for females and 4.06; 1.51 to 6.45 for males), but stable trends in adults of both sexes. We showed substantial increase in admissions for DUD across all strata, ranging from 5.47 (3.42 to 7.25) in male seniors to 10.26 (8.38 to 12.18) in female adolescents and emerging adults. Next, while admissions due to AUD were consistent with a stable trend in male adults (0.62; -0.03 to 1.28), they were rising in the remaining strata, with the most pronounced annual increases in female adolescents and emerging adults (5.96; 4.79 to 7.14). When considering schizophrenia, the admission rates were decreasing for female adults (-0.69; -1.03 to -0.35) and male seniors (-1.28; -2.49 to -0.08), but were stable in other strata. We detected that admissions for depression were consistent with a stable trend in female adolescents and emerging adults (0.11; -0.71 to 0.93) and male adults (-0.83; -1.75 to 0.08), whereas in the remaining strata, the trend was decreasing, most markedly in female seniors (-3.48; -4.38 to -2.70). Admission rates for anxiety disorders were increasing uniformly across all strata, ranging from 0.53 (0.03 to 1.04) in female adults to 3.26 (2.65 to 3.89) in male adolescents and emerging adults. For eating disorders, while admission rates were consistent with a stable trend for female and male adolescents and emerging adults (-0.20; -2.95 to 2.56 and 3.15; -0.34 to 6.56), we showed an increasing trend in the remaining strata, with the most pronounced increments in female seniors (6.54; 3.36 to 9.82). Finally, admissions due to personality disorders were increasing in female adolescents and emerging adults (3.38; 2.70 to 3.95), but decreasing in the remaining strata, most notably in male seniors (-7.93; -10.08 to -6.47). For detailed AAPCs see Supplementary Table 1, for APCs Fig. 1 and Supplementary Tables 4, 5, 6, and for identified breakpoints Supplementary Table 7.

### Median length of stay

**Fig. 2:**
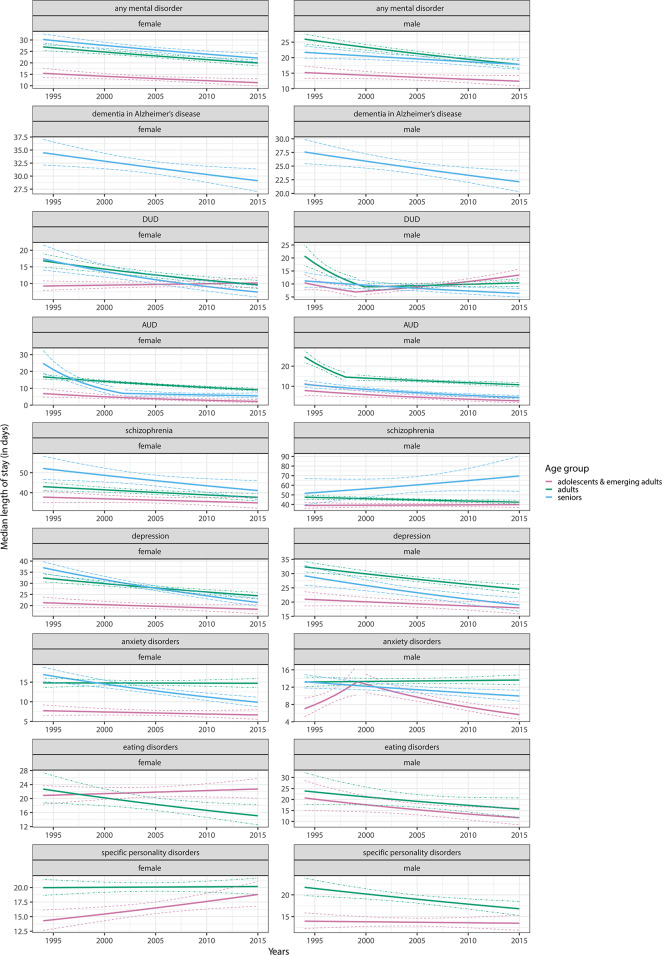
Age- and sex-specific temporal trends in median length of stay. The results are expressed as annual percent changes with 95% confidence intervals. “AUD” an “DUD” refer to alcohol use disorders and drug use disorders, respectively

The median LOS for any adult mental disorder decreased across all age and sex strata (e.g., AAPC = -1.43; 95% CI = -1.95 to -0.92 in female and  -1.78; -2.33 to -1.26 in male adults) except for male adolescents and emerging adults, for whom estimates were consistent with a stable trend (-0.96; -2.02 to 0.10). For dementia in Alzheimer’s disease, we identified decreases in median LOS in both female (-0.81; -1.39 to -0.22) and male (-1.04; -1.71 to -0.39) seniors. While the median LOS for DUD decreased for adults and seniors of both sexes, we detected an increase in male adolescents and emerging adults (1.23; 0.39 to 2.07) and estimates consistent with a stable trend in female adolescents and emerging adults (0.45; -0.77 to 1.67). For AUD, we showed substantial decreases across all strata, ranging from  -2.87 (-3.63 to -2.21) in female adults to -6.90 (-8.47 to -5.29) in female seniors. The median LOS for schizophrenia decreased slightly in adults of both sexes as well as female seniors, but was consistent with a stable trend in other strata. For depression, the median LOS decreased in adults and seniors of both sexes, increased in male adolescents and emerging adults (1.68; 0.17 to 3.21), and was consistent with a stable trend in female adolescents and emerging adults (-0.71; -1.62 to 0.19). Then, median LOS for anxiety disorders decreased in seniors of both sexes, but showed a stable trend in the remaining strata. We demonstrated decreases in median LOS for eating disorders in female adults (-1.93, -3.43 to -0.47) and male adolescents (-2.68, -5.33 to -0.12), but for other strata, the estimates were consistent with a stable trend. Finally, for personality disorders, the median LOS increased in female adolescents and emerging adults (1.32; 0.37 to 2.25), decreased in male adults (-1.22; -1.99 to -0.46), and was consistent with a stable trend in the remaining strata. For detailed AAPCs see Supplementary Table 2, for APCs Fig. 2 and Supplementary Tables 8, 9, 10, and for identified breakpoints Supplementary Table 11.

### Standardized inpatient-years

**Fig. 3:**
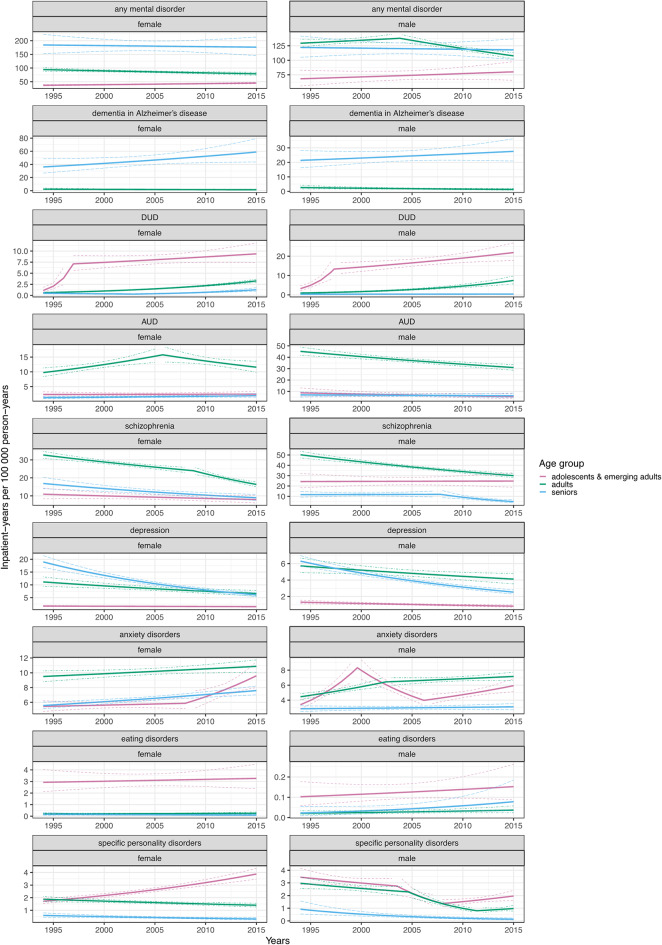
Age- and sex-specific temporal trends in inpatient-years. The results are expressed as annual percent changes with 95% confidence intervals. “AUD” an “DUD” refer to alcohol use disorders and drug use disorders, respectively

**Fig. 4:**
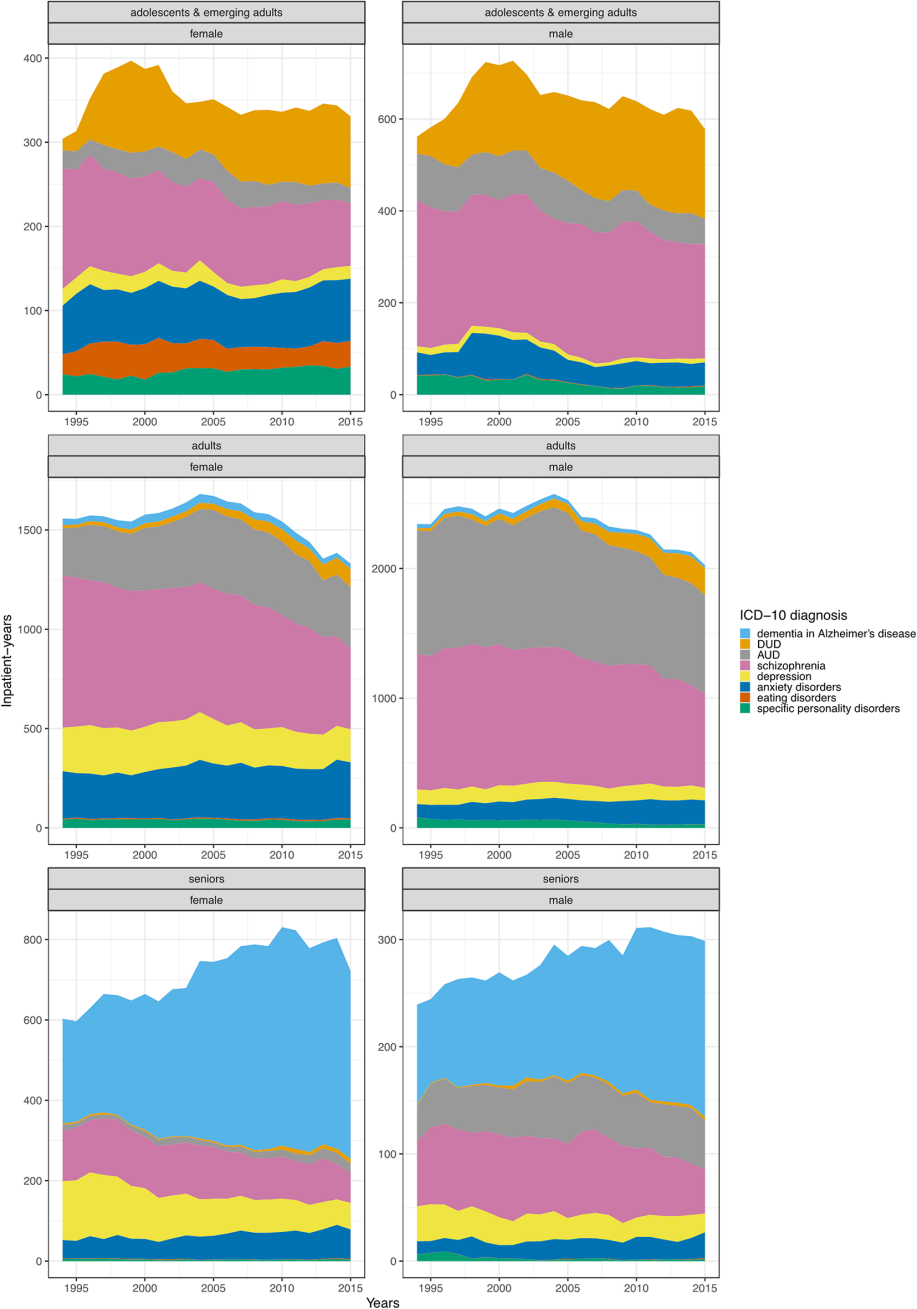
Age- and sex-specific temporal trends in composition of inpatient care use. The results indicate the number of inpatient-years per studied mental disorders throughout the examined time period. “AUD” an “DUD” refer to alcohol use disorders and drug use disorders, respectively

When considering any adult mental disorder, we showed reductions in SIY in adults of both sexes (AAPC = -0.85; 95% CI = -1.42 to -0.28 for females and  -0.87; -1.19 to -0.56 for males), increases in female adolescents and emerging adults (0.95; 0.42 to 1.47), and estimates consistent with a stable trend in the remaining strata. For dementia in Alzheimer’s disease, adults and seniors of both sexes demonstrated SIY estimates consistent with a stable trend. Next, while SIY for DUD was consistent with a stable trend in male seniors (1.12; -1.88 to 4.13), it was increasing in the remaining strata, with the most pronounced annual increases in female adolescents and emerging adults (10.68; 8.52 to 12.89) and male adults (10.41; 7.72 to 12.08). For AUD, the SIY decreased in male adults (-1.78; -2.45 to -1.14), but the estimates were consistent with a stable trend in the remaining strata. Next, while estimates of SIY for schizophrenia indicated a stable trend in adolescents and emerging adults, they decreased markedly in other strata, ranging from  -2.41 (-2.97 to -1.92) in male adults to -4.02 (-5.44, -2.57) in male seniors. We demonstrated decreasing SIY for depression in each studied strata, most markedly in female seniors (-5.25; -6.38 to -4.41), except for female adolescents and emerging adults for whom the estimates were consistent with a stable trend (-0.66; -1.82 to 0.49). Conversely, except for male seniors (0.41; -0.67 to 1.50), SIY for anxiety disorders were increasing, ranging from 0.64 (0.01 to 1.27) in female adults to 2.73 (1.66 to 3.81) in female adolescents and emerging adults. For eating disorders, SIY estimates across all the strata were consistent with a stable trend. Finally, whereas SIY for personality disorders increased in female adolescents and emerging adults (3.94; 3.00 to 4.73), it decreased in all other strata, ranging from  -1.38 (-2.25 to -0.53) in female adults to -8.80 (-13.06 to -5.36) in male seniors. For detailed AAPCs see Supplementary Table 3, for APCs Fig. 3 and Supplementary Tables 12, 13, 14, for identified breakpoints Supplementary Table 15, and for composition of inpatient care use Fig. 4.

## Discussion

### Main findings

In the present study, we described temporal trends in inpatient care use due to common adult mental disorders in Czechia in the time period from 1994 to 2015. Overall, the admission rates remained relatively unchanged in seniors, slightly increased in adults, whereas adolescents and emerging adults demonstrated a more profound increase. We demonstrated that the median LOS for adult mental disorders was decreasing, except for male adolescents and emerging adults who showed stable levels in time. Taken together, trends in admission rates and median LOS suggest that inpatient episodes for adult mental disorders have become more frequent and shorter over time. Then, the overall use of inpatient care, as assessed by SIY, showed reductions in adults, unchanged levels in seniors and male adolescents and emerging adults, but an increase in female adolescents and emerging adults. These findings are broadly in line with the observed trends in many Western countries in the last decades of the 20^th^ century and the beginning of the 21^st^ century [31–35].

### Potential mechanisms

Several mechanisms, most likely interacting with each other, may be responsible for the observed trends. First, notable changes in the provision of inpatient care occurred. Given the high costs of psychiatric hospitalizations [21] and deinstitutionalization trends elsewhere in Western Europe [31–37], the observed trends might have been caused by a growing policy emphasis on reductions of long-term hospitalizations, increased tendency towards cost-effectiveness, and changes in reimbursements. The number of beds in psychiatric hospitals was decreasing in Czechia, with 99 beds per 100,000 inhabitants in 1997 and 81 beds per 100,000 inhabitants in 2015 [45]. Moreover, the bed occupancy rates increased from 88.0% in 2000 to 93.3% in 2015 [45]. This suggests increasing pressure on effective resource utilization, thus possibly incentivizing clinicians to prefer earlier discharges or zero-day hospitalizations and to favour admissions for certain conditions.

Second, the number of outpatient psychiatrists per 100,000 inhabitants rose from 4.96 in 1996 to 8.30 in 2015 [45], potentially permitting a reduction in the length of hospitalization by enhanced aftercare. Also, the reductions in inpatient service use in people with schizophrenia might be related to the introduction of novel psychopharmacological treatments, such as atypical antipsychotics and long-lasting depot injections, which make outpatient treatment more feasible. Although we do not have data on trends of antipsychotic drug use in Czechia over the past decades, some evidence suggests significant increases in overall prescription drug expenditures over time concurrent with decreases in inpatient expenditures [55]. Next, antidepressant drug consumption in Czechia witnessed one of the largest increases in Europe between 2000 and 2015 [56]. Although this increase may, in part, reflect more overuse and off-label use [57], it is also likely to represent the aftercare of discharged hospital patients.

Third, societal changes brought on by the transition from a one-party political system and a centrally organized economy to an elected, capitalist democracy, need to be considered. This transition led to previously unknown societal challenges, including unemployment, that likely contributed to worsened mental health outcomes of the population in the 1990s. Similarly, after a period of relative obscurity before the Velvet Revolution, there was a profound increase in the availability of psychoactive substances in the 1990s transition period, leading to elevated levels of drug and alcohol use [58], and, consequently, to potential mental health harms associated with the use of these substances.

Additionally, other factors shared across the Western world, including (a) increased awareness about mental health and greater acceptance of professional help for mental health problems [59], (b) changes in the underlying population of people with mental disorders, particularly for internalizing disorders in female adolescents [60], and (c) the gradual proliferation of e-mental health and self-help services might have played a role.

### Potential implications of the observed trends

The allocation of resources did not seem to follow the patient discharges from inpatient care, with the financing of the outpatient sector remaining relatively stable, accounting for 15.3% and 14% of the mental health care budget in 2006 and 2015, respectively [42, 43]. A qualitative study investigating general practitioners’ and outpatient psychiatrists’ needs found that outpatient services are overloaded [61]. Moreover, community-based care remained to be insufficiently deployed in Czechia in the past decades, with services such as mobile crisis teams, day clinics, and residential housing remaining largely inaccessible due to low numbers and uneven distribution across the country [44]. Furthermore, in a treatment satisfaction study, only half of patients discharged from inpatient services obtained information on where to turn in case of additional health issues and/or other problems [44], suggesting a lack of patient monitoring and low implementation of integrated community care [62].

The number of people with schizophrenia treated in the outpatient sector demonstrated only a limited increase despite decreases in inpatient care use [45], raising concerns about the adequacy of provided care and assistance. Given the lack of community-based alternatives and the overloaded outpatient sector, the group of people with severe mental illnesses such as those with schizophrenia might have experienced an overall reduction in care upon their discharge, possibly deepening the existing treatment gap in Czechia [63]. Moreover, 14.9% of long-stay patients with psychosis in Czechia were shown to be re-hospitalized within 2 weeks after discharge from inpatient care [24], suggesting the presence of a revolving-door phenomenon. While the subsequent therapeutic pathways of the discharged individuals in Czechia are largely unknown, these issues are of serious concern and warrant further investigation.

### Limitations

The present study has some limitations. First, in some mental disorders, a number of age and sex combinations contained excessive zero counts (e.g., eating disorders in males), and we cannot rule out the possibility that we did not capture the magnitude of temporal trends optimally in these instances. In future investigations, segmented zero-inflated models might be more appropriate when the primary research interest lies in estimating temporal trends in strata with such rare outcome occurrences [64]. Second, mental disorders can also be listed as secondary diagnoses; however, we restricted our analyses to those listed as primary diagnoses. We did so to capture the actual cause of the inpatient stay, but we cannot rule out the possibility that some mental disorders were systematically more or less likely to be listed as the primary diagnosis throughout the studied time period. Third, in some instances, we found considerable age-specific heterogeneity in time trends; thus, a more fine-grained assessment considering age-specific developments (e.g., in older adolescents) is warranted. Fourth, in a number of European countries, there has been a broad trend of moving away from psychiatric hospitals as the dominant institutions, with general hospital-based mental health services growing in importance [10]. However, we did not distinguish between hospitalizations in general hospitals’ psychiatric wards and psychiatric hospitals, thus possibly masking differential patterns in time trends. Finally, although the ICD-10 taxonomy was used throughout the whole observation period, changes in recording and diagnostic practices may undermine the temporal consistency of the psychiatric diagnoses, biasing the identified time trends. However, we are not aware of research indicating significant confounding factors in diagnostic practice over the time period of this study.

## Conclusion

In the present study, we demonstratred stable or increasing trends for psychiatric hospital admissions coupled with considerable reductions in the length of stay in Czechia from 1994 to 2015. The overall psychiatric inpatient care use was decreasing or stable in adults and seniors, suggesting a gradual shift away from hospital-based care. Future research investigating subsequent individual trajectories of people discharged from psychiatric inpatient care is warranted to evaluate the success of deinstitutionalization efforts and to monitor the mental health care provision.

## Electronic supplementary material

Below is the link to the electronic supplementary material.

Supplementary Material 1

## Data Availability

Due to its sensitive character, the data cannot be published or shared with external subjects without a permission granted by the Czech Institute of Health Information and Statistics. L.P., P.W. and T.F. had full access to all data in the study. L.P. and T.F. take responsibility for the integrity of the data and the accuracy of the data analysis. The full analytical code is available at a dedicated GitHub repository: https://github.com/libpot/Time_Trends_Psych_Hosp_Czechia.

## References

[1] Plana-Ripoll O, Pedersen CB, Agerbo E et al (2019) A comprehensive analysis of mortality-related health metrics associated with mental disorders: a nationwide, register-based cohort study. Lancet 394:1827–1835. 10.1016/S0140-6736(19)32316-5 31668728 10.1016/S0140-6736(19)32316-5

[2] Walker ER, McGee RE, Druss BG (2015) Mortality in mental disorders and global disease burden implications. JAMA Psychiatry 72:334–341. 10.1001/jamapsychiatry.2014.2502 25671328 10.1001/jamapsychiatry.2014.2502 PMC4461039

[3] Global Burden of Disease Study 2016 (2016) Data Resources | GHDx. https://ghdx.healthdata.org/gbd-2016. Accessed 27 Jan 2023

[4] Rehm J, Shield KD (2019) Global burden of disease and the impact of mental and addictive disorders. Curr Psychiatry Rep 21:10. 10.1007/s11920-019-0997-0 30729322 10.1007/s11920-019-0997-0

[5] Vos T, Lim SS, Abbafati C et al (2020) Global burden of 369 diseases and injuries in 204 countries and territories, 1990–2019: a systematic analysis for the global burden of Disease Study 2019. Lancet 396:1204–1222. 10.1016/S0140-6736(20)30925-9 33069326 10.1016/S0140-6736(20)30925-9 PMC7567026

[6] Olesen J, Gustavsson A, Svensson M et al (2012) The economic cost of brain disorders in Europe. Eur J Neurol 19:155–162. 10.1111/j.1468-1331.2011.03590.x 22175760 10.1111/j.1468-1331.2011.03590.x

[7] Drew N, Funk M, Tang S et al (2011) Human rights violations of people with mental and psychosocial disabilities: an unresolved global crisis. Lancet 378:1664–1675. 10.1016/S0140-6736(11)61458-X 22008426 10.1016/S0140-6736(11)61458-X

[8] Turner T (2004) The history of deinstitutionalization and reinstitutionalization. Psychiatry 3:1–4. 10.1383/psyt.3.9.1.50257

[9] Yohanna D (2013) Deinstitutionalization of people with mental illness: causes and consequences. AMA J Ethics 15:886–891. 10.1001/virtualmentor.2013.15.10.mhst1-1310 10.1001/virtualmentor.2013.15.10.mhst1-1310 24152782

[10] Becker T, Vázquez-Barquero JL (2001) The European perspective of psychiatric reform. Acta Psychiatrica Scandinavica 104:8–14. 10.1034/j.1600-0447.2001.1040s2008.x 10.1034/j.1600-0447.2001.1040s2008.x 11863056

[11] Gilburt H, Peck E, Ashton B et al (2014) Service transformation lessons from mental health. The King’s Fund

[12] Haug H-J, Rössler W (1999) Deinstitutionalization of psychiatric patients in central Europe. Eur Archives Psychiatry Clin Neurosciences 249:115–122. 10.1007/s004060050075 10.1007/s004060050075 10433124

[13] Knapp M, Beecham J, McDaid D et al (2011) The economic consequences of deinstitutionalisation of mental health services: lessons from a systematic review of European experience. Health Soc Care Commun 19:113–125. 10.1111/j.1365-2524.2010.00969.x 10.1111/j.1365-2524.2010.00969.x 21143545

[14] Thornicroft G, Alem A, Antunes Dos Santos R et al (2010) WPA guidance on steps, obstacles and mistakes to avoid in the implementation of community mental health care. World Psychiatry 9:67–77. 10.1002/j.2051-5545.2010.tb00276.x 20671888 10.1002/j.2051-5545.2010.tb00276.x PMC2911080

[15] Thornicroft G, Tansella M (2013) The balanced care model: the case for both hospital- and community-based mental healthcare. Br J Psychiatry 202:246–248. 10.1192/bjp.bp.112.111377 23549938 10.1192/bjp.bp.112.111377

[16] World Health Organization. Regional Office for Europe (2015) The European mental health action plan 2013–2020. World Health Organization. Regional Office for Europe

[17] Häfner H, Heiden W, an der (1989) The evaluation of mental health care systems. Br J Psychiatry 155:12–17. 10.1192/bjp.155.1.12 2513999 10.1192/bjp.155.1.12

[18] Knapp M, McDaid AA D, et al (2014) Investing in recovery. Making the business case for effective interventions for people with schizophrenia and psychosis

[19] Reinharz D, Lesage AD, Contandriopoulos AP (2000) Cost-effectiveness analysis of psychiatric deinstitutionalization. Can J Psychiatry 45:533–538. 10.1177/070674370004500603 10986570 10.1177/070674370004500603

[20] Winkler P, Broulíková HM, Kondrátová L et al (2018) Value of schizophrenia treatment II: decision modelling for developing early detection and early intervention services in the Czech Republic. Eur Psychiatry 53:116–122. 10.1016/j.eurpsy.2018.06.008 30036774 10.1016/j.eurpsy.2018.06.008

[21] Winkler P, Koeser L, Kondrátová L et al (2018) Cost-effectiveness of care for people with psychosis in the community and psychiatric hospitals in the Czech Republic: an economic analysis. Lancet Psychiatry 5:1023–1031. 10.1016/S2215-0366(18)30388-2 30415938 10.1016/S2215-0366(18)30388-2

[22] Bird V, Premkumar P, Kendall T et al (2010) Early intervention services, cognitive–behavioural therapy and family intervention in early psychosis: systematic review. Br J Psychiatry 197:350–356. 10.1192/bjp.bp.109.074526 21037211 10.1192/bjp.bp.109.074526 PMC2966501

[23] Randall JR, Vokey S, Loewen H et al (2015) A systematic review of the effect of early interventions for psychosis on the usage of Inpatient services. Schizophr Bull 41:1379–1386. 10.1093/schbul/sbv016 25745034 10.1093/schbul/sbv016 PMC4601703

[24] Winkler P, Mladá K, Krupchanka D et al (2016) Long-term hospitalizations for schizophrenia in the Czech Republic 1998–2012. Schizophr Res 175:180–185. 10.1016/j.schres.2016.04.008 27094718 10.1016/j.schres.2016.04.008

[25] Furlan PM, Zuffranieri M, Stanga F et al (2009) Four-year Follow-Up of Long-Stay patients settled in the Community after Closure of Italy’s Psychiatric hospitals. PS 60:1198–1202. 10.1176/ps.2009.60.9.1198 10.1176/ps.2009.60.9.1198 19723734

[26] Kunitoh N (2013) From hospital to the community: the influence of deinstitutionalization on discharged long-stay psychiatric patients. J Neuropsychiatry Clin Neurosci 67:384–396. 10.1111/pcn.12071 10.1111/pcn.12071 23890091

[27] Thornicroft G, Bebbington P, Leff J (2005) Outcomes for long-term patients one year after Discharge from a Psychiatric Hospital. PS 56:1416–1422. 10.1176/appi.ps.56.11.1416 10.1176/appi.ps.56.11.1416 16282261

[28] Babalola O, Gormez V, Alwan NA et al (2014) Length of hospitalisation for people with severe mental illness. Cochrane Database Syst Reviews. 10.1002/14651858.CD000384.pub3 10.1002/14651858.CD000384.pub3 PMC10105316 24477710

[29] Johnstone P, Zolese G (1999) Systematic review of the effectiveness of planned short hospital stays for mental health care. BMJ 318:1387–1390. 10.1136/bmj.318.7195.1387 10334748 10.1136/bmj.318.7195.1387 PMC27881

[30] Winkler P, Barrett B, McCrone P et al (2016) Deinstitutionalised patients, homelessness and imprisonment: systematic review. Br J Psychiatry 208:421–428. 10.1192/bjp.bp.114.161943 27143007 10.1192/bjp.bp.114.161943

[31] Goldman HH, Adams NH, Taube CA (1983) Deinstitutionalization: the Data Demythologized. PS 34:129–134. 10.1176/ps.34.2.129 10.1176/ps.34.2.129 6860396

[32] Lay B, Nordt C, Rössler W (2007) Trends in psychiatric hospitalisation of people with schizophrenia: a register-based investigation over the last three decades. Schizophr Res 97:68–78. 10.1016/j.schres.2007.07.006 17689930 10.1016/j.schres.2007.07.006

[33] Salize HJ, Rössler W, Becker T (2007) Mental health care in Germany. Eur Arch Psychiatry Clin Neurosci 257:92–103. 10.1007/s00406-006-0696-9 17149540 10.1007/s00406-006-0696-9

[34] Levinson D, Lerner Y, Lichtenberg P (2003) Reduction in inpatient length of stay and changes in mental health care in Israel over four decades: a national case register study. Isr J Psychiatry Relat Sci 40:240–247 14971125

[35] Saz-Parkinson Z, Medel A, Cediel-García P et al (2011) Trends on schizophrenia admissions during the deinstitutionalisation process in Spain (1980–2004). Soc Psychiatry Psychiatr Epidemiol 46:1095–1101. 10.1007/s00127-010-0289-9 20972771 10.1007/s00127-010-0289-9

[36] Burti L (2001) Italian psychiatric reform 20 plus years after. Acta Psychiatr Scand Suppl 41–46. 10.1034/j.1600-0447.2001.1040s2041.x 10.1034/j.1600-0447.2001.1040s2041.x 11863050

[37] Barbato A (1998) Psychiatry in Transition: outcomes of Mental Health Policy Shift in Italy. Aust N Z J Psychiatry 32:673–679. 10.3109/00048679809113122 9805590 10.3109/00048679809113122

[38] Forsman AK, Ventus DBJ, van der Feltz-Cornelis CM et al (2014) Public mental health research in Europe: a systematic mapping for the ROAMER project. Eur J Public Health 24:955–960. 10.1093/eurpub/cku055 25428662 10.1093/eurpub/cku055

[39] Krupchanka D, Winkler P (2016) State of mental healthcare systems in Eastern Europe: do we really understand what is going on? BJPsych Int 13:96–99 29093919 10.1192/s2056474000001446 PMC5619493

[40] Pec O (2019) Mental health reforms in the Czech Republic. BJPsych Int 16:4–6. 10.1192/bji.2017.27 30747157 10.1192/bji.2017.27 PMC6357523

[41] Winkler P, Krupchanka D, Roberts T et al (2017) A blind spot on the global mental health map: a scoping review of 25 years’ development of mental health care for people with severe mental illnesses in central and eastern Europe. Lancet Psychiatry 4:634–642. 10.1016/S2215-0366(17)30135-9 28495549 10.1016/S2215-0366(17)30135-9

[42] Broulikova HM, Dlouhy M, Winkler P (2020) Expenditures on Mental Health Care in the Czech Republic in 2015. Psychiatr Q 91:113–125. 10.1007/s11126-019-09688-3 31773471 10.1007/s11126-019-09688-3 PMC7033065

[43] Dlouhy M (2011) Mental health services in the health accounts: the Czech Republic. Soc Psychiatry Psychiatr Epidemiol 46:447–453. 10.1007/s00127-010-0210-6 20300727 10.1007/s00127-010-0210-6

[44] Höschl C, Winkler P, Pěč O (2012) The state of psychiatry in the Czech Republic. Int Rev Psychiatry 24:278–285. 10.3109/09540261.2012.688730 22950765 10.3109/09540261.2012.688730

[45] Psychiatrická péče 2016 - ÚZIS ČR. https://www.uzis.cz/index.php?pg=record&id=7920. Accessed 21 Feb 2023

[46] Ministry of Health of the Czech Republic (2013) Strategy for the reform of psychiatric care. Ministry of Health of the Czech Republic

[47] Bryndová L, Šlegerová L, Votápková J et al (2023) Czechia: Health System Review. Health Syst Transit 25:1–216 36951272

[48] Degli Esposti M, Ziauddeen H, Bowes L et al (2022) Trends in inpatient care for psychiatric disorders in NHS hospitals across England, 1998/99-2019/20: an observational time series analysis. Soc Psychiatry Psychiatr Epidemiol 57:993–1006. 10.1007/s00127-021-02215-5 34951652 10.1007/s00127-021-02215-5 PMC8705084

[49] Arnett JJ, Žukauskienė R, Sugimura K (2014) The new life stage of emerging adulthood at ages 18–29 years: implications for mental health. Lancet Psychiatry 1:569–576. 10.1016/S2215-0366(14)00080-7 26361316 10.1016/S2215-0366(14)00080-7

[50] OECD (2018) Length of hospital stay. Organisation for Economic Co-operation and Development, Paris

[51] Muggeo VMR (2003) Estimating regression models with unknown break-points. Stat Med 22:3055–3071. 10.1002/sim.1545 12973787 10.1002/sim.1545

[52] Tiwari RC, Cronin KA, Davis W et al (2005) Bayesian model selection for join point regression with application to age-adjusted cancer rates. J Roy Stat Soc: Ser C (Appl Stat) 54:919–939. 10.1111/j.1467-9876.2005.00518.x

[53] Clegg LX, Hankey BF, Tiwari R et al (2009) Estimating average annual per cent change in trend analysis. Stat Med 28:3670–3682. 10.1002/sim.3733 19856324 10.1002/sim.3733 PMC2843083

[54] (2023) R Core Team (2021). R: A language and environment for statistical computing. R Foundation for Statistical Computing, Vienna, Austria. URL https://www.R-project.org/. https://www.r-project.org/. Accessed 27 Jan 2023

[55] Miller LS, Martin BC (2004) Current and future forecasts of service use and expenditures of Medicaid-eligible schizophrenia patients in the state of Georgia. Schizophr Bull 30:983–995. 10.1093/oxfordjournals.schbul.a007147 15954202 10.1093/oxfordjournals.schbul.a007147

[56] OECD (2017) Health at a glance 2017: OECD indicators. Organisation for Economic Co-operation and Development, Paris

[57] Wong J, Motulsky A, Abrahamowicz M et al (2017) Off-label indications for antidepressants in primary care: descriptive study of prescriptions from an indication based electronic prescribing system. BMJ 356:j603. 10.1136/bmj.j603 28228380 10.1136/bmj.j603 PMC5320934

[58] Csémy L, Kubička L, Nociar A (2002) Drug scene in the Czech republic and slovakia during the period of transformation. Eur Addict Res 8:159–165 12457055 10.1159/000066134

[59] Schomerus G, Schwahn C, Holzinger A et al (2012) Evolution of public attitudes about mental illness: a systematic review and meta-analysis. Acta Psychiatrica Scandinavica 125:440–452. 10.1111/j.1600-0447.2012.01826.x 22242976 10.1111/j.1600-0447.2012.01826.x

[60] Keyes KM, Platt JM (2024) Annual Research Review: sex, gender, and internalizing conditions among adolescents in the 21st century - trends, causes, consequences. J Child Psychol Psychiatry 65:384–407. 10.1111/jcpp.13864 37458091 10.1111/jcpp.13864 PMC12341061

[61] Stuchlík J, Wenigová B (2008) Vzdělávací potřeby praktických lékařů a ambulantních psychiatrů v oblasti péče o duševně nemocné. Medicína pro praxi 4:519–520

[62] Mohr P, Galderisi S, Boyer P et al (2018) Value of schizophrenia treatment I: the patient journey. Eur Psychiatry 53:107–115. 10.1016/j.eurpsy.2018.06.007 30036773 10.1016/j.eurpsy.2018.06.007

[63] Kagstrom A, Alexova A, Tuskova E et al (2019) The treatment gap for mental disorders and associated factors in the Czech Republic. Eur Psychiatry 59:37–43. 10.1016/j.eurpsy.2019.04.003 31009916 10.1016/j.eurpsy.2019.04.003

[64] Dourado P, Pedroso-de-Lima AC, Rocha FMM (2023) Segmented zero-inflated Poisson. mixed effects model with random changepoint

