# Supplementary material for: Temporal trends in inpatient care use for adult mental disorders in Czechia: a nationwide register-based study from 1994 to 2015
Source: Soc Psychiatry Psychiatr Epidemiol. 2024 May 31;59(10):1825–37. doi: 10.1007/s00127-024-02691-5 (PMC11464556; doi:10.1007/s00127-024-02691-5)
Supplement: Supplementary file 1 — Supplementary Material 1 [file 127_2024_2691_MOESM1_ESM.docx]

**Temporal trends in inpatient care use for adult mental disorders in Czechia: A nationwide register-based study from 1994 to 2015**

Libor Potočár, Petr Winkler, Pavel Mohr, Tomáš Formánek

**Supplementary Materials**

Table of Contents

[Supplementary Table 1 Age- and sex-specific temporal trends in admissions to inpatient care expressed as average annual percent change 4](#_Toc165291901)

[Supplementary Table 2 Age- and sex-specific temporal trends in median length of stay expressed as average annual percent change 5](#_Toc165291902)

[Supplementary Table 3 Age- and sex-specific temporal trends in inpatient-years expressed as average annual percent change 6](#_Toc165291903)

[Supplementary Table 4 Sex-specific temporal trends trends in admissions to inpatient care in adolescents and emerging adults expressed as annual percent change 7](#_Toc165291904)

[Supplementary Table 5 Sex-specific temporal trends trends in admissions to inpatient care in adults expressed as annual percent change 8](#_Toc165291905)

[Supplementary Table 6 Sex-specific temporal trends trends in admissions to inpatient care in seniors expressed as annual percent change 9](#_Toc165291906)

[Supplementary Table 7 Age- and sex-specific breakpoints in admissions to inpatient care 10](#_Toc165291907)

[Supplementary Table 8 Sex-specific temporal trends trends in median length of stay in adolescents and emerging adults expressed as annual percent change 12](#_Toc165291908)

[Supplementary Table 9 Sex-specific temporal trends trends in median length of stay in adults expressed as annual percent change 13](#_Toc165291909)

[Supplementary Table 10 Sex-specific temporal trends trends in median length of stay in seniors expressed as annual percent change 14](#_Toc165291910)

[Supplementary Table 11 Age- and sex-specific breakpoints in median length of stay 15](#_Toc165291911)

[Supplementary Table 12 Sex-specific temporal trends trends in inpatient-years in adolescents and emerging adults expressed as annual percent change 16](#_Toc165291912)

[Supplementary Table 13 Sex-specific temporal trends trends in inpatient-years in adults expressed as annual percent change 17](#_Toc165291913)

[Supplementary Table 14 Sex-specific temporal trends trends in inpatient-years in seniors expressed as annual percent change 18](#_Toc165291914)

[Supplementary Table 15 Age- and sex-specific breakpoints in inpatient-years 19](#_Toc165291915)

# Supplementary Table 1 Age- and sex-specific temporal trends in admissions to inpatient care expressed as average annual percent change

| Diagnosis | Adolescents &  emerging adults | | Adults | | Seniors | |
| --- | --- | --- | --- | --- | --- | --- |
|  | females | males | females | males | females | males |
| Any mental disorder | 3.27 (2.57,3.97) | 2.98 (2.08,3.88) | 0.51 (0.16,0.86) | 1.01 (0.63,1.4) | 1.22  (-0.31,2.73) | 1.35  (-0.3,2.98) |
| Dementia in Alzheimer’s disease | NA | NA | -1.79  (-5.69,2.09) | -2.76  (-6.81,1.22) | 4.08 (1.45,6.55) | 4.06  (1.51,6.45) |
| DUD | 10.26 (8.38,12.18) | 7.46 (6.24,8.68) | 9.01 (7.77,9.49) | 9.27 (6.66,11.09) | 6.98 (5.83,8.14) | 5.47  (3.42,7.25) |
| AUD | 5.96 (4.79,7.14) | 2.26 (1.21,3.26) | 2.16 (1.17,3.15) | 0.62  (-0.03,1.28) | 4.88 (3.12,6.41) | 3.27  (1.71,4.74) |
| Schizophrenia | -0.08  (-1.97,1.8) | 2.06  (-0.13,4.22) | -0.69  (-1.03,-0.35) | -0.48  (-1.28,0.32) | -0.41  (-1.9,1.08) | -1.28  (-2.49,-0.08) |
| Depression | 0.11  (-0.71,0.93) | -1.06  (-2.02,-0.1) | -1.45  (-2.53,-0.4) | -0.83  (-1.75,0.08) | -3.48  (-4.38,-2.7) | -2.2  (-2.76,-1.69) |
| Anxiety disorders | 2.69  (1.62,3.7) | 3.26 (2.65,3.89) | 0.53 (0.03,1.04) | 1.9 (1.26,2.52) | 2.42 (1.76,3.04) | 2.3  (1.33,3.27) |
| Eating disorders | -0.2  (-2.95,2.56) | 3.15  (-0.34,6.56) | 2.86 (0.65,4.99) | 4.26 (2.04,6.33) | 6.54 (3.36,9.82) | 6.54 (1.78,11.06) |
| Specific personality disorders | 3.38  (2.7,3.95) | -1.99  (-2.88,-1.1) | -1.19  (-2.12,-0.27) | -4.41  (-5.88,-3.15) | -3.43  (-4.94,-2.03) | -7.93  (-10.08,-6.47) |

The results are expressed as average annual percent change with 95% confidence intervals. NA denotes situations when the estimation was not performed. “AUD” an “DUD” refer to alcohol use disorders and drug use disorders, respectively. Adolescents and emerging adults were aged from 15 to 29 years, adults were aged from 30 to 64 years, and seniors were aged 65 or more years.

# Supplementary Table 2 Age- and sex-specific temporal trends in median length of stay expressed as average annual percent change

| Diagnosis | Adolescents &  emerging adults | | Adults | | Seniors | |
| --- | --- | --- | --- | --- | --- | --- |
|  | females | males | females | males | females | males |
| Any mental disorder | -1.45  (-2.54,-0.38) | -0.96  (-2.02,0.1) | -1.43  (-1.95,-0.92) | -1.78  (-2.33,-1.26) | -1.48  (-2.14,-0.84) | -0.94  (-1.69,-0.2) |
| Dementia in Alzheimer’s disease | NA | NA | NA | NA | -0.81  (-1.39,-0.22) | -1.04  (-1.71,-0.39) |
| DUD | 0.45  (-0.77,1.67) | 1.23 (0.39,2.07) | -2.63  (3.66,-1.67) | -3.21  (-4.21,-2.21) | -3.99  (-5.86,-2.28) | -2.63  (-4.76,-0.58) |
| AUD | -5.28  (-8.57,-2.34) | -4.88  (-8.08,-1.96) | -2.87  (-3.63,-2.21) | -3.86  (-4.21,-3.51) | -6.9  (-8.47,-5.29) | -4.23  (-5.74,-2.91) |
| Schizophrenia | -0.37  (-1.01,0.26) | 0.11  (-0.5,0.71) | -0.63  (-1.02,-0.24) | -0.55  (-0.93,-0.18) | -1.13  (-2.05,-0.23) | 1.44  (-0.7,3.55) |
| Depression | -0.71  (-1.62,0.19) | 1.68 (0.17,3.21) | -1.34  (-1.82,-0.88) | -1.3  (-1.78,-0.84) | -2.58  (-3.23,-1.99) | -2.03  (-3.02,-1.09) |
| Anxiety disorders | -0.73  (-2.15,0.69) | -1.03  (-2.17,0.13) | -0.03  (-0.68,0.62) | 0.16  (-0.52,0.84) | -2.54  (-3.49,-1.65) | -1.35  (-2.34,-0.39) |
| Eating disorders | 0.41  (-0.59,1.4) | -2.68  (-5.33,-0.12) | -1.93  (-3.43,-0.47) | -1.98  (-4.27,0.25) | NA | NA |
| Specific personality disorders | 1.32 (0.37,2.25) | -0.17  (-1.19,0.86) | 0.05  (-0.51,0.6) | -1.22  (-1.99,-0.46) | NA | NA |

The results are expressed as average annual percent change with 95% confidence intervals. NA denotes situations when the estimation was not performed. “AUD” an “DUD” refer to alcohol use disorders and drug use disorders, respectively. Adolescents and emerging adults were aged from 15 to 29 years, adults were aged from 30 to 64 years, and seniors were aged 65 or more years.

# Supplementary Table 3 Age- and sex-specific temporal trends in inpatient-years expressed as average annual percent change

| Diagnosis | Adolescents &  emerging adults | | Adults | | Seniors | |
| --- | --- | --- | --- | --- | --- | --- |
|  | females | males | females | males | females | males |
| Any mental disorder | 0.95 (0.42,1.47) | 0.77  (-0.83,2.37) | -0.85  (-1.42,-0.28) | -0.87  (-1.19,-0.56) | -0.21  (-1.79,1.37) | -0.17  (-1.41,1.07) |
| Dementia in Alzheimer’s disease | NA | NA | -2.26  (-7.2,2.64) | -3.29  (-7.16,0.48) | 2.35  (-0.16,4.8) | 1.22  (-0.99,3.42) |
| DUD | 10.68 (8.52,12.89) | 9.56 (7.75,11.4) | 8.26 (7.1,8.76) | 10.41 (7.72,12.08) | 4.8  (2.9,6.73) | 1.12  (-1.88,4.13) |
| AUD | 0.21  (-2.56,2.98) | -2.2  (-5.4,0.94) | 0.82  (-0.14,1.78) | -1.78  (-2.45,-1.14) | 2.07  (-0.38,4.48) | -0.54  (-3.24,2.16) |
| Schizophrenia | -1.51  (-3.6,0.56) | 0.1  (-2.11,2.31) | -3.25  (-3.68,-2.82) | -2.41  (-2.97,-1.92) | -2.97  (-4.58,-1.46) | -4.02  (-5.44,-2.57) |
| Depression | -0.66  (-1.82,0.49) | -2.16  (-3.48,-0.87) | -2.44  (-3.82,-1.13) | -1.56  (-2.82,-0.32) | -5.25  (-6.38,-4.41) | -4.25  (-5.15,-3.53) |
| Anxiety disorders | 2.73 (1.66,3.81) | 2.71 (1.93,3.49) | 0.64 (0.01,1.27) | 2.3 (1.74,2.85) | 1.48 (0.84,2.11) | 0.41  (-0.67,1.5) |
| Eating disorders | 0.53  (-2.02,3.09) | 1.9  (-2.35,6.17) | 1.24  (-1.5,3.97) | 2.67  (-0.87,6.12) | -2.8  (-7.68,2.12) | 6.07  (-1.46,13.34) |
| Specific personality disorders | 3.94  (3.00,4.73) | -2.64  (-3.71,-1.56) | -1.38  (-2.25,-0.53) | -5.16  (-6.19,-4.11) | -2.95  (-5.22,-0.77) | -8.8  (-13.06,-5.36) |

The results are expressed as average annual percent change with 95% confidence intervals. NA denotes situations when the estimation was not performed. “AUD” an “DUD” refer to alcohol use disorders and drug use disorders, respectively. Adolescents and emerging adults were aged from 15 to 29 years, adults were aged from 30 to 64 years, and seniors were aged 65 or more years.

# Supplementary Table 4 Sex-specific temporal trends trends in admissions to inpatient care in adolescents and emerging adults expressed as annual percent change

| Diagnosis | Females | | | | | | Males | | | | | |
| --- | --- | --- | --- | --- | --- | --- | --- | --- | --- | --- | --- | --- |
|  | APC  1 | Time period 1 | APC  2 | Time period  2 | APC 3 | Time period 3 | APC  1 | Time period 1 | APC  2 | Time period 2 | APC  3 | Time period 3 |
| Any mental disorder | 9.91  (2.79, 17.51) | 1994 - 1997 | 2.02  (1.33,2.71) | 1997 - 2015 | NA | NA | 14.75  (7.63,22.35) | 1994 - 1998 | 0.31  (0.7,1.32) | 1998 - 2015 | NA | NA |
| DUD | 70.21  (43.65,101.68) | 1994 - 1997 | 1.56  (-0.15,3.3) | 1997 - 2015 | NA | NA | 46.02  (30.58,63.29) | 1994 - 1997 | 0.12  (-1,1.25) | 1997 - 2015 | NA | NA |
| AUD | 10.15  (6.67,13.75) | 1994 - 2003 | 2.29  (-0.14,4.77) | 2003 - 2015 | NA | NA | NA | NA | NA | NA | NA | NA |
| Schizophrenia | NA | NA | NA | NA | NA | NA | NA | NA | NA | NA | NA | NA |
| Depression | NA | NA | NA | NA | NA | NA | NA | NA | NA | NA | NA | NA |
| Anxiety disorders | NA | NA | NA | NA | NA | NA | 16.62 (13,20.36) | 1994 - 1999 | -6.47  (-8.76,4.12) | 1999 - 2006 | 4.21  (2.45,6.01) | 2006 - 2015 |
| Eating disorders | NA | NA | NA | NA | NA | NA | NA | NA | NA | NA | NA | NA |
| Specific personality disorders | NA | NA | NA | NA | NA | NA | 2.67  (-0.02,5.43) | 1994 - 2002 | -14.21  (-19.78,  - 8.24) | 2002 - 2007 | 3.33  (0.13,6.92) | 2007 - 2015 |

“APC” refers to annual percent change and it is presented with 95% confidence intervals. NA denotes situtations when there were no breakpoints. “AUD” an “DUD” refer to alcohol use disorders and drug use disorders, respectively. Adolescents and emerging adults were aged from 15 to 29 years.

# Supplementary Table 5 Sex-specific temporal trends trends in admissions to inpatient care in adults expressed as annual percent change

| Diagnosis | Females | | | | | | Males | | | | | |
| --- | --- | --- | --- | --- | --- | --- | --- | --- | --- | --- | --- | --- |
|  | APC  1 | Time period 1 | APC  2 | Time period  2 | APC 3 | Time period 3 | APC  1 | Time period 1 | APC  2 | Time period 2 | APC  3 | Time period 3 |
| Any mental disorder | NA | NA | NA | NA | NA | NA | 3.59  (2.04,5.16) | 1994 - 2001 | -0.35  (-0.99,0.3) | 2001 - 2015 | NA | NA |
| Dementia in Alzheimer’s disease | NA | NA | NA | NA | NA | NA | NA | NA | NA | NA | NA | NA |
| DUD | NA | NA | NA | NA | NA | NA | NA | NA | NA | NA | NA | NA |
| AUD | 6.51  (3.1,10.03) | 1994 - 2002 | -0.69  (-2.52,1.18) | 2002 - 2015 | NA | NA | 5.17  (2.5,7.92) | 1994 - 2001 | -1.84 (-2.92,-0.75) | 2001 - 2015 | NA | NA |
| Schizophrenia | NA | NA | NA | NA | NA | NA | NA | NA | NA | NA | NA | NA |
| Depression | NA | NA | NA | NA | NA | NA | NA | NA | NA | NA | NA | NA |
| Anxiety disorders | NA | NA | NA | NA | NA | NA | NA | NA | NA | NA | NA | NA |
| Eating disorders | NA | NA | NA | NA | NA | NA | NA | NA | NA | NA | NA | NA |
| Specific personality disorders | NA | NA | NA | NA | NA | NA | NA | NA | NA | NA | NA | NA |

“APC” refers to annual percent change and it is presented with 95% confidence intervals. NA denotes situtations when there were no breakpoints. “AUD” an “DUD” refer to alcohol use disorders and drug use disorders, respectively. Adults were aged from 30 to 64 years.

# Supplementary Table 6 Sex-specific temporal trends trends in admissions to inpatient care in seniors expressed as annual percent change

| Diagnosis | Females | | | | | | Males | | | | | |
| --- | --- | --- | --- | --- | --- | --- | --- | --- | --- | --- | --- | --- |
|  | APC  1 | Time period 1 | APC  2 | Time period  2 | APC 3 | Time period 3 | APC  1 | Time period 1 | APC  2 | Time period 2 | APC  3 | Time period 3 |
| Any mental disorder | NA | NA | NA | NA | NA | NA | NA | NA | NA | NA | NA | NA |
| Dementia in Alzheimer’s disease | NA | NA | NA | NA | NA | NA | NA | NA | NA | NA | NA | NA |
| DUD | 3.3  (0.66,6.01) | 1994 - 2005 | 25.44 (16.44,35.13) | 2005 - 2010 | 1.59  (4.18,7.71) | 2010 - 2015 | NA | NA | NA | NA | NA | NA |
| AUD | NA | NA | NA | NA | NA | NA | NA | NA | NA | NA | NA | NA |
| Schizophrenia | NA | NA | NA | NA | NA | NA | NA | NA | NA | NA | NA | NA |
| Depression | NA | NA | NA | NA | NA | NA | NA | NA | NA | NA | NA | NA |
| Anxiety disorders | NA | NA | NA | NA | NA | NA | 5.25  (2.36,8.22) | 1994 - 2003 | -0.3  (-2.25,1.69) | 2003 - 2015 | NA | NA |
| Eating disorders | -2.22  (-9.12,5.21) | 1994 - 2005 | 19.45  (9.44,30.38) | 2005 - 2015 | NA | NA | NA | NA | NA | NA | NA | NA |
| Specific personality disorders | NA | NA | NA | NA | NA | NA | NA | NA | NA | NA | NA | NA |

“APC” refers to annual percent change and it is presented with 95% confidence intervals. NA denotes situtations when there were no breakpoints. “AUD” an “DUD” refer to alcohol use disorders and drug use disorders, respectively. Seniors were aged 65 or more years.

# Supplementary Table 7 Age- and sex-specific breakpoints in admissions to inpatient care

| Diagnosis | Breakpoint  number | Adolescents &  emerging adults | | Adults | | Seniors | |
| --- | --- | --- | --- | --- | --- | --- | --- |
|  |  | females | males | females | males | females | males |
| Any mental disorder | 1 | 1997.43 (1995.17, 1999.69) | 1998.10 (1996.65, 1999.56) | NA | 2001.33 (1999.11, 2003.56) | NA | NA |
| DUD | 1 | 1997.34 (1996.55, 1998.14) | 1997.93 (1997.07, 1998.79) | NA | NA | 2006.00 (2004.58, 2007.42) | NA |
| DUD | 2 | NA | NA | NA | NA | 2010.20 (2008.98, 2011.41) | NA |
| AUD | 1 | 2004.00 (2000.53, 2007.47) | NA | 2002.48 (1999.32, 2005.63) | 2001.54 (1999.36, 2003.71) | NA | NA |
| Schizophrenia | 1 | NA | NA | NA | NA | NA | NA |
| Depression | 1 | NA | NA | NA | NA | NA | NA |
| Anxiety disorders | 1 | NA | 1999.05 (1998.37, 1999.72) | NA | NA | NA | 2003.96 (1999.98, 2007.93) |
| Anxiety disorders | 2 | NA | 2006.07 (2004.83, 2007.32) | NA | NA | NA | NA |
| Eating disorders | 1 | NA | NA | NA | NA | 2006.00 (2002.26, 2009.74) | NA |
| Specific personality disorders | 1 | NA | 2002.00 (2000.56, 2003.44) | NA | NA | NA | NA |
| Specific personality disorders | 2 | NA | 2007.69 (2006.28, 2009.10) | NA | NA | NA | NA |

The results are expressed as breakpoints (in calendar years) with 95% confidence intervals. NA denotes situtations when there were no breakpoints. “AUD” an “DUD” refer to alcohol use disorders and drug use disorders, respectively. Adolescents and emerging adults were aged from 15 to 29 years, adults were aged from 30 to 64 years, and seniors were aged 65 or more years.

# Supplementary Table 8 Sex-specific temporal trends trends in median length of stay in adolescents and emerging adults expressed as annual percent change

| Diagnosis | Females | | | | | | Males | | | | | |
| --- | --- | --- | --- | --- | --- | --- | --- | --- | --- | --- | --- | --- |
|  | APC  1 | Time period 1 | APC  2 | Time period  2 | APC 3 | Time period 3 | APC  1 | Time period 1 | APC  2 | Time period 2 | APC  3 | Time period 3 |
| Any mental disorder | NA | NA | NA | NA | NA | NA | NA | NA | NA | NA | NA | NA |
| DUD | NA | NA | NA | NA | NA | NA | -7.88  (-12.64,-2.87) | 1994 - 1999 | 4.25  (3.09, 5.42) | 1999 - 2015 | NA | NA |
| AUD | NA | NA | NA | NA | NA | NA | NA | NA | NA | NA | NA | NA |
| Schizophrenia | NA | NA | NA | NA | NA | NA | NA | NA | NA | NA | NA | NA |
| Depression | NA | NA | NA | NA | NA | NA | 49.16  (7.37, 107.21) | 1994 - 1995 | -2.68  (-4.06,-1.27) | 1995 - 2010 | 7.96  (0.17, 16.36) | 2010 - 2015 |
| Anxiety disorders | NA | NA | NA | NA | NA | NA | 13.19  (6.62, 20.16) | 1994 - 1999 | -5.25  (-6.61,-3.88) | 1999 - 2015 | NA | NA |
| Eating disorders | NA | NA | NA | NA | NA | NA | NA | NA | NA | NA | NA | NA |
| Specific personality disorders | NA | NA | NA | NA | NA | NA | NA | NA | NA | NA | NA | NA |

“APC” refers to annual percent change and it is presented with 95% confidence intervals. NA denotes situtations when there were no breakpoints. “AUD” an “DUD” refer to alcohol use disorders and drug use disorders, respectively. Adolescents and emerging adults were aged from 15 to 29 years.

# Supplementary Table 9 Sex-specific temporal trends trends in median length of stay in adults expressed as annual percent change

| Diagnosis | Females | | | | | | Males | | | | | |
| --- | --- | --- | --- | --- | --- | --- | --- | --- | --- | --- | --- | --- |
|  | APC  1 | Time period 1 | APC  2 | Time period  2 | APC 3 | Time period 3 | APC  1 | Time period 1 | APC  2 | Time period 2 | APC  3 | Time period 3 |
| Any mental disorder | NA | NA | NA | NA | NA | NA | NA | NA | NA | NA | NA | NA |
| DUD | NA | NA | NA | NA | NA | NA | -13.48  (-18.55,-8.09) | 1994 - 1999 | 1.09  (-0.44,2.65) | 1999 - 2015 | NA | NA |
| AUD | NA | NA | NA | NA | NA | NA | -12.26  (-14.53,-9.93) | 1994 - 1998 | -1.76  (-2.26,-1.26) | 1998 - 2015 | NA | NA |
| Schizophrenia | NA | NA | NA | NA | NA | NA | NA | NA | NA | NA | NA | NA |
| Depression | NA | NA | NA | NA | NA | NA | NA | NA | NA | NA | NA | NA |
| Anxiety disorders | NA | NA | NA | NA | NA | NA | NA | NA | NA | NA | NA | NA |
| Eating disorders | NA | NA | NA | NA | NA | NA | NA | NA | NA | NA | NA | NA |
| Specific personality disorders | NA | NA | NA | NA | NA | NA | NA | NA | NA | NA | NA | NA |

“APC” refers to annual percent change and it is presented with 95% confidence intervals. NA denotes situtations when there were no breakpoints. “AUD” an “DUD” refer to alcohol use disorders and drug use disorders, respectively. Adults were aged from 30 to 64 years.

# Supplementary Table 10 Sex-specific temporal trends trends in median length of stay in seniors expressed as annual percent change

| Diagnosis | Females | | | | | | Males | | | | | |
| --- | --- | --- | --- | --- | --- | --- | --- | --- | --- | --- | --- | --- |
|  | APC  1 | Time period 1 | APC  2 | Time period  2 | APC 3 | Time period 3 | APC  1 | Time period 1 | APC  2 | Time period 2 | APC  3 | Time period 3 |
| Any mental disorder | NA | NA | NA | NA | NA | NA | NA | NA | NA | NA | NA | NA |
| Dementia in Alzheimer’s disease | NA | NA | NA | NA | NA | NA | NA | NA | NA | NA | NA | NA |
| DUD | NA | NA | NA | NA | NA | NA | NA | NA | NA | NA | NA | NA |
| AUD | -15  (-20.3,-9.36) | 1994 - 2001 | -1.75  (-4.93,1.54) | 2001 - 2015 | NA | NA | NA | NA | NA | NA | NA | NA |
| Schizophrenia | NA | NA | NA | NA | NA | NA | NA | NA | NA | NA | NA | NA |
| Depression | NA | NA | NA | NA | NA | NA | NA | NA | NA | NA | NA | NA |
| Anxiety disorders | NA | NA | NA | NA | NA | NA | NA | NA | NA | NA | NA | NA |

“APC” refers to annual percent change and it is presented with 95% confidence intervals. NA denotes situtations when there were no breakpoints. “AUD” an “DUD” refer to alcohol use disorders and drug use disorders, respectively. Seniors were aged 65 or more years.

# Supplementary Table 11 Age- and sex-specific breakpoints in median length of stay

| Diagnosis | Breakpoint  number | Adolescents &  emerging adults | | Adults | | Seniors | |
| --- | --- | --- | --- | --- | --- | --- | --- |
|  |  | females | males | females | males | females | males |
| Any mental disorder | 1 | NA | NA | NA | NA | NA | NA |
| DUD | 1 | NA | 1999.00 (1997.30, 2000.70) | NA | 1999.87 (1998.08, 2001.66) | NA | NA |
| AUD | 1 | NA | NA | NA | 1998.02 (1997.25, 1998.79) | 2001.80 (1998.88, 2004.71) | NA |
| Schizophrenia | 1 | NA | NA | NA | NA | NA | NA |
| Depression | 1 | NA | 1995.00 (1994.44, 1995.56) | NA | NA | NA | NA |
| Depression | 2 | NA | 2010.25 (2007.57, 2012.93) | NA | NA | NA | NA |
| Anxiety disorders | 1 | NA | 1999.15 (1997.95, 2000.36) | NA | NA | NA | NA |
| Eating disorders | 1 | NA | NA | NA | NA | NA | NA |
| Specific personality disorders | 1 | NA | NA | NA | NA | NA | NA |

The results are expressed as breakpoints (in calendar years) with 95% confidence intervals. NA denotes situtations when there were no breakpoints. “AUD” an “DUD” refer to alcohol use disorders and drug use disorders, respectively. Adolescents and emerging adults were aged from 15 to 29 years, adults were aged from 30 to 64 years, and seniors were aged 65 or more years.

# Supplementary Table 12 Sex-specific temporal trends trends in inpatient-years in adolescents and emerging adults expressed as annual percent change

| Diagnosis | Females | | | | | | Males | | | | | |
| --- | --- | --- | --- | --- | --- | --- | --- | --- | --- | --- | --- | --- |
|  | APC  1 | Time period 1 | APC  2 | Time period  2 | APC 3 | Time period 3 | APC  1 | Time period 1 | APC  2 | Time period 2 | APC  3 | Time period 3 |
| Any mental disorder | NA | NA | NA | NA | NA | NA | NA | NA | NA | NA | NA | NA |
| DUD | 86.73 (39.87,149.27) | 1994 - 1996 | 1.55  (-0.17,3.3) | 1996 - 2015 | NA | NA | 53.62 (30.34,81.05) | 1994 - 1997 | 2.83 (1.13,4.57) | 1997 - 2015 | NA | NA |
| AUD | NA | NA | NA | NA | NA | NA | NA | NA | NA | NA | NA | NA |
| Schizophrenia | NA | NA | NA | NA | NA | NA | NA | NA | NA | NA | NA | NA |
| Depression | NA | NA | NA | NA | NA | NA | NA | NA | NA | NA | NA | NA |
| Anxiety disorders | 0.54  (-1.02,2.12) | 1994 - 2008 | 7.26 (2.08,12.7) | 2008 - 2015 | NA | NA | 17.4 (12.8,22.19) | 1994 - 1999 | -10.68 (-13.46,-7.81) | 1999 - 2006 | 4.65 (2.41,6.93) | 2006 - 2015 |
| Eating disorders | NA | NA | NA | NA | NA | NA | NA | NA | NA | NA | NA | NA |
| Specific personality disorders | NA | NA | NA | NA | NA | NA | -2.36  (-5.17,0.54) | 1994 - 2003 | -14.38  (-23.98,-3.57) | 2003 - 2007 | 5.29 (1.05,9.7) | 2007 - 2015 |

“APC” refers to annual percent change and it is presented with 95% confidence intervals. NA denotes situtations when there were no breakpoints. “AUD” an “DUD” refer to alcohol use disorders and drug use disorders, respectively. Adolescents and emerging adults were aged from 15 to 29 years.

# Supplementary Table 13 Sex-specific temporal trends trends in inpatient-years in adults expressed as annual percent change

| Diagnosis | Females | | | | | | Males | | | | | |
| --- | --- | --- | --- | --- | --- | --- | --- | --- | --- | --- | --- | --- |
|  | APC  1 | Time period 1 | APC  2 | Time period  2 | APC 3 | Time period 3 | APC  1 | Time period 1 | APC  2 | Time period 2 | APC  3 | Time period 3 |
| Any mental disorder | NA | NA | NA | NA | NA | NA | 0.65  (-0.27,1.59) | 1994 - 2003 | -2.19  (-2.87,-1.5) | 2003 - 2015 | NA | NA |
| Dementia in Alzheimer’s disease | NA | NA | NA | NA | NA | NA | NA | NA | NA | NA | NA | NA |
| DUD | NA | NA | NA | NA | NA | NA | NA | NA | NA | NA | NA | NA |
| AUD | 4.16  (2.01,6.36) | 1994 - 2005 | -3.29  (-5.91,-0.59) | 2005 - 2015 | NA | NA | NA | NA | NA | NA | NA | NA |
| Schizophrenia | -2.08  (-2.73,-1.42) | 1994 - 2008 | -6  (-7.96,-4) | 2008 - 2015 | NA | NA | NA | NA | NA | NA | NA | NA |
| Depression | NA | NA | NA | NA | NA | NA | NA | NA | NA | NA | NA | NA |
| Anxiety disorders | NA | NA | NA | NA | NA | NA | 4.5  (2.6,6.43) | 1994 - 2002 | 0.85  (-0.2,1.92) | 2002 - 2015 | NA | NA |
| Eating disorders | NA | NA | NA | NA | NA | NA | NA | NA | NA | NA | NA | NA |
| Specific personality disorders | NA | NA | NA | NA | NA | NA | -2.41  (-4.52,-0.26) | 1994 - 2004 | -14.46  (-18.08,  -10.67) | 2004 - 2011 | 5.61  (4.68,17.02) | 2011 - 2015 |

“APC” refers to annual percent change and it is presented with 95% confidence intervals. NA denotes situtations when there were no breakpoints. “AUD” an “DUD” refer to alcohol use disorders and drug use disorders, respectively. Adults were aged from 30 to 64 years.

# Supplementary Table 14 Sex-specific temporal trends trends in inpatient-years in seniors expressed as annual percent change

| Diagnosis | Females | | | | | | Males | | | | | |
| --- | --- | --- | --- | --- | --- | --- | --- | --- | --- | --- | --- | --- |
|  | APC  1 | Time period 1 | APC  2 | Time period  2 | APC 3 | Time period 3 | APC  1 | Time period 1 | APC  2 | Time period 2 | APC  3 | Time period 3 |
| Any mental disorder | NA | NA | NA | NA | NA | NA | NA | NA | NA | NA | NA | NA |
| Dementia in Alzheimer’s disease | NA | NA | NA | NA | NA | NA | NA | NA | NA | NA | NA | NA |
| DUD | -5.15  (-10.8,0.87) | 1994 - 2002 | 12.93  (9.02,16.98) | 2002 - 2015 | NA | NA | NA | NA | NA | NA | NA | NA |
| AUD | NA | NA | NA | NA | NA | NA | NA | NA | NA | NA | NA | NA |
| Schizophrenia | NA | NA | NA | NA | NA | NA | 0.18  (-2.33,2.75) | 1994 - 2007 | -11.57  (-16.64,-6.19) | 2007 - 2015 | NA | NA |
| Depression | NA | NA | NA | NA | NA | NA | NA | NA | NA | NA | NA | NA |
| Anxiety disorders | NA | NA | NA | NA | NA | NA | NA | NA | NA | NA | NA | NA |
| Eating disorders | NA | NA | NA | NA | NA | NA | NA | NA | NA | NA | NA | NA |
| Specific personality disorders | NA | NA | NA | NA | NA | NA | NA | NA | NA | NA | NA | NA |

“APC” refers to annual percent change and it is presented with 95% confidence intervals. NA denotes situtations when there were no breakpoints. “AUD” an “DUD” refer to alcohol use disorders and drug use disorders, respectively. Seniors were aged 65 or more years.

# Supplementary Table 15 Age- and sex-specific breakpoints in inpatient-years

| Diagnosis | Breakpoint  number | Adolescents &  emerging adults | | Adults | | Seniors | |
| --- | --- | --- | --- | --- | --- | --- | --- |
|  |  | females | males | females | males | females | males |
| Any mental disorder | 1 | NA | NA | NA | 2003.80 (2001.24, 2006.36) | NA | NA |
| DUD | 1 | 1996.97 (1995.89, 1998.04) | 1997.31 (1996.32, 1998.31) | NA | NA | 2003.00 (2000.52, 2005.48) | NA |
| AUD | 1 | NA | NA | 2005.76 (2002.86, 2008.66) | NA | NA | NA |
| Schizophrenia | 1 | NA | NA | 2008.80 (2006.35, 2011.25) | NA | NA | 2007.80 (2005.09, 2010.50) |
| Depression | 1 | NA | NA | NA | NA | NA | NA |
| Anxiety disorders | 1 | 2008.00 (2003.97, 2012.03) | 1999.61 (1998.90, 2000.31) | NA | 2002.41 (1998.92, 2005.90) | NA | NA |
| Anxiety disorders | 2 | NA | 2006.16 (2005.08, 2007.24) | NA | NA | NA | NA |
| Specific personality disorders | 1 | NA | 2003.52 (2001.04, 2006.00) | NA | 2004.63 (2002.94, 2006.32) | NA | NA |
| Specific personality disorders | 2 | NA | 2008.00 (2006.18, 2009.82) | NA | 2011.36 (2009.93, 2012.80) | NA | NA |

The results are expressed as breakpoints (in calendar years) with 95% confidence intervals. NA denotes situtations when there were no breakpoints. “AUD” an “DUD” refer to alcohol use disorders and drug use disorders, respectively. Adolescents and emerging adults were aged from 15 to 29 years, adults were aged from 30 to 64 years, and seniors were aged 65 or more years.
